
JOURNAL INFORMATION
==============================
NLM Title Abbreviation: J Can Health Libr Assoc
Journal ID: J Can Health Libr Assoc
NLM Journal ID: 101229936
Journal ID: JCHLA

The Journal of the Canadian Health Libraries Association

EISSN: 1708-6892
Publisher: Canadian Health Libraries Association / Association des bibliotèques de la santé du Canada

ARTICLE INFORMATION
==============================
PMCID: PMC10403116
DOI: 10.29173/jchla29720
Article ID: JCHLA-44-048
Article version: 1
Subjects: Abstracts

CHLA 2023 CONFERENCE CONTRIBUTED PAPERS / ABSC CONGRÈS 2023 COMMUNICATIONS LIBRES

Electronic publication date: 2023 Aug 1
Publication date: 2023 Aug
Volume: 44
Issue: 2
First page: 48
Last page: 63
License: This article is distributed under a Creative Commons Attribution License: https://creativecommons.org/licenses/by/4.0/
License URL: https://creativecommons.org/licenses/by/4.0/

==============================

## CP1. Exploring the impact of automated indexing on completeness of MeSH terms

Alexandre Amar-Zifkin, Virginie Paquet, Taline Ekmekjian, Tara Landry
Université de Montréal

**Introduction:** The use of controlled vocabulary to identify relevant articles is a central element of bibliographic database instruction in health sciences. Students learning to search MEDLINE are taught that MeSH yields precise results, and that MeSH indexing increases an article's findability, reliably describing an article's contents. Indexing for MEDLINE was done completely by human indexers until 2011. Since April 2022, all articles are assigned MeSH via automated indexing (AI). Per the NLM, MeSH assigned by AI are determined based on terms in title, abstract, and terms and indexing of 'related records', with human review and curation of results "as appropriate". As MEDLINE instruction typically starts with teaching learners to identify key elements or concepts in their research question and find appropriate MeSH for them, we sought to explore the following: how well does AI identify key concepts of an article? Are concepts missed more or less when compared to human indexers? Drawing on the PICO framework, are missing concepts more often any particular PICO element? **Methods:** We reviewed samples of automated and human-indexed records from shortly before April 2022, and some entirely-automated from later, to determine whether their main concepts were adequately represented with MeSH. Working in pairs, our team used a web form to assign key concepts (based on the PICO framework) that, per our experience, would be used to find it and similar articles based on title and abstract. Assigned MeSH were then displayed and analyzed to determine whether they adequately represented the key concepts of each record. **Results & Conclusion:** As the study is ongoing, results are forthcoming. Potential impacts of Automated Indexing on library instruction and basic searching will be discussed.

## CP2. Can GPT-3 tools accurately find and analyze articles for systematic reviews? A (very) preliminary assessment

Gary Atwood
University of Vermont

**Introduction:** GPT-3 is a large language model that uses artificial intelligence to generate textual responses to prompts and questions. GPT-3 technology has been used to create several interesting tools including the widely reported chatbot ChatGPT-3, which was released in November 2022. Inspired by the initial success of GPT-3, several organizations have started to build tools designed to assist with tasks associated with the systematic review research process. This project will analyze how successful these tools are in completing two specific tasks: searching for research articles and analyzing individual articles. **Methods/Description:** This project consists of two parts. In part one, the research question from a previously published systematic review will be used to conduct a search in two GPT-3 based tools for relevant research articles. In part two, each GPT-3 tool will be used to analyze a single research article to determine if it is relevant to the research question **Results/outcomes:** For part one, the results will be based on how effective and efficient each tool is at finding relevant research articles. Results will be compared to the set of articles included in the original review as a measure of success. For part two, each tool will be used to pull evaluative information from the sample article. This information will be compared to a manual assessment completed by the author. **Discussion:** This project will provide researchers with guidance on how to integrate GPT-3 based tools into their systematic review workflow. It will include a brief discussion of strengths and weaknesses and how they can impact potential results.

## CP3. Preliminary results of a longitudinal study of health information-seeking behaviour pre- and post-COVID

Joan Bartlett, Aaron Bowen-Ziecheck
McGill University

**Introduction:** As we pass three years since the declaration of the COVID-19 pandemic, the question persists as to how 'normal' has evolved since then. Recent research into health information-seeking behaviour (ISB) has found evidence of differences related to the COVID-19 pandemic. Our pre-pandemic research into undergraduate students' ISB identified a mismatch between the rating of a resource's credibility, and the frequency of its use. In contrast, since the pandemic started, students report taking a more critical approach to selecting and appraising health information, including checking information against other sources, verifying if information is up-to-date, and assessing the author/institution to check for bias. Here, we present a longitudinal study of health ISB before and after the pandemic started to determine: 1) if/how health ISB has changed from before the pandemic, and 2) whether health ISB differs for everyday health issues, and those specifically related to COVID-19. **Methods:** Data collection involves two online surveys of McGill University undergraduate students, the first before the pandemic, and a replicate survey in early 2023. The pre-pandemic survey (in the context of everyday health issues) yielded over 3500 responses. The follow-up survey includes the same questions regarding frequency of resource use, and assessment of resource credibility. The questions are repeated for two contexts: everyday health issues, and specifically related to COVID-19. **Results:** Data collection and analysis are in progress; preliminary results of the post-pandemic survey, with comparison to the pre-pandemic survey will be presented. **Discussion:** We will discuss the implications of the preliminary findings for health librarianship, with focus on how health ISB has evolved during the pandemic, and the implications for the 'new normal' of the future.

## CP4. Librarian teaching practices in support of knowledge syntheses projects: Preliminary results of an environmental scan

**Introduction:** Previous program descriptions describe that Health Sciences librarians teach search skills for knowledge synthesis (KS) projects. Yet, little research discusses instructional practices and approaches. We conducted an environmental scan to address this gap. Our questions were: Do health science librarians teach searching methods for KS projects in groups of three or more learners? For those that do, what tools and approaches are employed? **Methods:** Following ethics approval, we developed a survey that included closed-ended, scaled, and open-ended responses. After piloting and incorporating feedback from three librarians, the online survey was distributed through social media and email lists. The survey was open to any librarian working directly with health sciences students and faculty. **Results:** We received 114 total responses to our survey from 10 different countries, with most respondents coming from Canada and the United States. This presentation will focus on the quantitative data analyzed using descriptive statistics, including topics covered and approaches for instructional delivery and assessment. The results reflect an emphasis on actionable searching skills in librarian instruction, but also inclusion of conceptual ideas and other steps of the review process. Analysis of results is ongoing and a selection of the results will be shared. **Discussion:** These selected results will be of interest to health library and information professionals wanting to know more about instructional practices used in group settings by librarians to help learners develop comprehensive searching skills for KS projects. We will discuss the results in the context of pedagogical practices and adult learning theories.

### CP5. Putting a sustainable systematic review into action

Sunny Chung, Laurel Scheinfeld, Jessica Koos, Jamie Saragossi
Stony Brook University

**Introduction:** The Stony Brook University Health Sciences Library has been supporting systematic reviews for a number of years, however, no formal policies or workflows were in place. There has also been a high number of uncompleted reviews, and no triaging system for distribution. A systematic review task force was established to create formal documents and policies, and streamline workflows so any librarian can step in and continue the review process. **Methods/Description:** Prior to meeting with a librarian, research teams are encouraged to complete the intake form so the librarians can determine who will take on the project. After the initial meeting, the team leader and librarian will sign a memorandum of understanding so that both parties understand their responsibilities before the search strategy is created. Next, the researcher(s) will need to complete the protocol template. To gauge the effectiveness of this new model, librarian satisfaction will be examined as well as the rate of project completion in the future. **Results/Outcomes:** The newly created systematic review service has fostered a better understanding and collaboration between the librarian and the research team. The new triaging system has formalized the internal workflow, resulting in increased awareness of ongoing systematic review projects among the librarian team. The adoption of a project management tool has facilitated efficient time management and allocation of resources. The health sciences librarians plan to continue using the newly implemented documents and policies as an integral part of our workflow. The systematic review task force will continue to monitor trends and modify these documents as needed.

### CP6. An environmental scan of librarian involvement in systematic reviews and scoping reviews at the University of Ottawa

Peter Farrell, Victoria Cole, Emily DaSilva, Valentina Ly, Nicholas Dehler, Alain El Hofi, Evan Sterling, Téa Rokolj, Sarah Visintini, Patrick Labelle, Lindsay Sikora,
University of Ottawa

**Introduction:** Requests for librarian collaboration on knowledge synthesis projects at the University of Ottawa have increased over time. Despite having significant impact on workload, the intellectual contributions of librarians are not always recognized through authorship or acknowledgement. We conducted an environmental scan to establish a baseline for how librarian involvement is reported in all systematic and scoping reviews published by authors affiliated with the University of Ottawa. Specifically, we wanted to better understand the publication volume, workload implications, and extent of reported librarian involvement. **Methods:** We searched 18 databases across various subject areas. Keyword and publication-type searches were combined with affiliation searches for the University of Ottawa or affiliated research institutes/centres. Published systematic reviews, scoping reviews, meta-analyses, and published protocols for these methodologies were included. All other review methodologies were excluded. Data extraction focused on bibliographic information, librarian involvement, funding, uOttawa affiliation, and the type of review. (Protocol available here: https://osf.io/bqd26/) **Results:** Search results yielded 20,270 records. After duplicates were removed, 8,805 records were screened by title and abstract. 6,049 records passed to full-text screening (currently underway). Preliminary results show reviews from a greater diversity of disciplines than originally anticipated. Consistency of review nomenclature varies greatly between disciplines and poses a challenge in ensuring consistency during screening. **Discussion:** This environmental scan maps systematic and scoping review publications across disciplines at the University of Ottawa. Better understanding the proportion of reviews with librarian involvement will help guide policy and procedure development around knowledge synthesis services, and enable us to project resourcing needs in the future.

## CP7. Advocating online for better knowledge translation in New Brunswick and beyond during the COVID-19 pandemic: I tried.

Kathleen Gadd; Cheryl Johnson

This paper is a description of a non-traditional program, accessible to all in English and a bit of French, undertaken virtually from my home while I cared for three young children during the first three years of the COVID-19 pandemic. Health sciences librarians recognized a need for information during the pandemic. As an independent librarian no longer working for an institution, I started a social media account and eventually an advocacy group to help others find high-quality information, and to disseminate the information provincial Public Health was declining to share. The failure of our governments to recognize airborne (inhalation) transmission of SARS-CoV-2 has been one of the most significant tragedies of the pandemic. As one librarian, and then as part of a team of multidisciplinary volunteers, I tried - we tried - to make a difference in the province of New Brunswick and beyond by engaging as many people as possible with links to high-quality citations, webinars, and other sources of evidence during a time of dangerous disinformation by various forces - and cruel silence by our provincial governments, who declined to inform departments, healthcare providers, or the public about airborne transmission, long COVID, and other vital findings in the literature. Without clear communication about these findings, without effective knowledge translation, people in New Brunswick and across Canada suffered needlessly. It didn't have to be this way. We tried. So far, knowledge alone hasn't been enough to effect systemic change. This paper details our efforts; making our work public in this way serves as a record that some fought back (with citations!) against mass infection - we hope it inspires, too.

## CP8. Perceptions and behaviours of first year health sciences undergraduate students conducting online research

Susanna Galbraith
McMaster University

**Objectives:** To explore the perceptions and behaviours of first year health sciences undergraduate students when conducting online research during their transition from secondary school into university. The aim of this research is to explore creative solutions to meeting the needs of today's pandemic affected students and their unique perceptions and behaviours. **Methods:** An ethics-board approved qualitative ethnographic study is underway using semi-structured interviews. Students were recruited from an introductory first year health sciences course. They were screened to ensure they are first year students and have no prior higher education experience. 13 students were successfully recruited. These students provided a 1-hour interview using Zoom during the first weeks of their undergraduate degree in the fall of 2022. Transcriptions have been verified by the principal investigator. The data is currently being analyzed using inductive coding methods, and will undergo multiple rounds of coding. Following this a survey will be developed and sent to participants in early 2023 to learn how their experience has changed over time. **Results:** Initial results are very preliminary at the time of this submission. The students interviewed described in detail all aspects of their online research process including: how they made decisions when searching Google; whether they tended to go to peers, instructors or online communities for support; when and why they would turn to YouTube; determining credibility of resources identified through Google; information literacy instruction they received during their secondary school years. **Conclusions:** The data is currently being analyzed, however much can be learned from this study. The conclusions will help to inform information literacy instruction for health sciences undergraduates, collection development and user experience design of library websites.

### CP9. Advocating for space in a hospital: one library's experience

Sarah Gleeson, Ashley Lavoie
Fraser Health

Hospital Libraries are always having to justify their existence within a hospital. With Covid-19 adding additional spacing requirements, our library was closed to facilitate increased social distancing for staff. In our search for a new space within the hospital, many lessons were learned along the way. Finding a new space for a library proved more difficult than the administration anticipated. In the design, physical limitations, such as weight restrictions, air quality, and noise levels needed to be met. Space is always at a premium in a hospital environment and there are many different stakeholders within a single hospital. Our move required navigating all the different players, offered spaces, and building requirements. Through reflection and user feedback, we were able to identify three key aspects that our hospital library required. We needed library office space, physical materials space, and also most importantly a community space. These three pieces became pivotal to our library designs. Library staff were able to liaise with multiple stakeholders for advocacy and design support. The language used in the residency accreditation was also key in communicating the value that library services provides to the hospital. Cooperative development was crucial in moving the project forward. Moving a library is never a simple task; however, by working with stakeholders that have shared values, we were able to create a solution that allowed us to open a library that is sustainable for the future.

### CP10. Factors and outcomes of collaboration in keeping up to date: An exploratory multiple case study

**Introduction:** Keeping up to date is fundamental for acquiring new knowledge and informing future action. Yet, it remains a challenge. Faced with information overload, researchers, information professionals, and health professionals have little time to continuously monitor and filter scholarly publications. This is particularly true for complex multidisciplinary fields like Patient Oriented Research (POR). Using collaboration to share the monitoring effort among group members may be a solution. The present study explored the factors and outcomes of collaborative information monitoring from the perspective of POR stakeholders using a dedicated information monitoring system. **Methods:** A multiple case study was conducted. Each monitoring project implemented in the system constituted a case. Data were collected using individual semi-structured interviews with researchers, research professionals, and health professionals. Interview data were corroborated with project documents and system logs. Data were analysed using deductive-inductive thematic analysis in two stages, within and across cases. **Results:** Seven cases were included. Results supported seven types of factors (personal, group, organizational, informational environment, information sources, system, task) and five types of outcomes (performance, behavioural, cognitive, affective, relational), which were identified in previous research. New sub-types were generated, 19 for factors and 12 for outcomes. **Discussion:** This research is innovative as it extends knowledge on collaboration in information monitoring. It offers actionable recommendations useful for implementing, supporting, and evaluating collaborative information projects, potentially helping POR stakeholders, as well as professionals in other fields, keep up to date. Moreover, the findings can inform the development of future information services and tools to support collaborative information monitoring, as well as information professionals' own collaborative projects.

## CP11. Learning on the job: Using artificial intelligence and natural language processing to support rapid review methods

Leah Hagerman, Sarah Neil-Sztramko, Maureen Dobbins,
The National Collaborating Centre for Methods and Tools

**Introduction:** The Rapid Evidence Service (RES) responds to the needs of public health decision-makers by answering priority questions using rapid review methods. The vast quantity of literature available makes it challenging to identify relevant research using manual screening under rapid review timelines. Our team has integrated Artificial Intelligence (AI) to support screening for rapid reviews. **Methods:** Four AI features offered by DistillerSR have been integrated into our screening methods: DAISY Rank, which uses Natural Language Processing to learn manual screening patterns and apply those to the remaining references to be screened, thereby predicting which studies will be most relevant; Re-Rank Report, which predicts the total number of included studies based on previous screening patterns; AI Screening, which automatically screens studies based on prediction scores; and Check for Screening Errors, which identifies studies that were potentially falsely excluded. **Results:** We have used DAISY Rank on 30 rapid reviews on 18 topics, allowing full text screening and data extraction to proceed by identifying the most relevant studies early. Use of the Re-Rank Report and Check for Screening Errors functions have informed decisions to move to AI Screening. To date, we have integrated AI Screening into five rapid reviews, including three living reviews. In one rapid review update, AI Screening automatically excluded 5,744 of 7,196 studies, thus saving time in manual screening. **Discussion:** Integrating AI into rapid review methods has saved time spent on manual screening, allowing reviews to progress faster. Our methods are transferable across most RES topics.

## CP12. Methods to support evidence-informed decision-making in public health: creation and evolution of a rapid review service

Leah Hagerman, Choudhry Nayab, Sarah Neil-Sztramko, Emily Clark, Susan Snelling, Robyn Traynor, Maureen Dobbins
The National Collaborating Centre for Methods and Tools

**Introduction:** The explosion of research literature that occurred in response to the COVID-19 pandemic resulted in the need for public health decision-makers to access high-quality synthesized evidence for policy and practice decisions. Our team quickly responded to the needs of decision makers by developing the Rapid Evidence Service (RES). The RES builds upon internationally accepted rapid review methodologies to synthesize the best available evidence on priority COVID-19 public health questions for local, regional and national public health decision makers. **Methods:** Our process involves: receiving and prioritizing public health decision makers' questions, collaboratively developing clearly-focused questions; developing and conducting a comprehensive search; rapid screening of evidence; critically appraising relevant evidence using validated tools; summarizing key findings; GRADE-ing the evidence; and synthesizing the findings into a final report which includes an executive summary. To ensure the needs of those most affected by subsequent decisions are represented, public partners are invited to provide their perspectives and lived experience. **Results:** Since May 2020, we have completed 82 rapid reviews on 44 priority public health topics. We continue to update reviews as new evidence emerges through both updates and maintaining living rapid reviews. We have answered questions from and worked collaboratively with regional, national, and international organizations. **Discussion:** The rapid review process is a real-world example of how review-level evidence can be mobilized quickly in response to decision-makers' needs. Our process has evolved to ensure feasibility, accuracy, and efficiency in response to and support recovery from the COVID-19 pandemic.

### CP13. Finding our way in a time of uncertainty: collaboration and the COVID-19 Hub

Joanne Hodder[1], Michelle Helliwell[1], Kate Mercer[1], Chelsey Millen[1], Noella Whelan[1], Ashley Daurie[1], Leah MacDonald[1], Katie McLean[1], Katie Puxley[2]
[1]Nova Scotia Health Authority; [2]Nova Scotia College of Art & Design

**Introduction:** In March 2020, the rapidity of internal documentation being created and released due to COVID-19 was straining the health authority's existing content management systems. **Description:** Staff showed signs of information overload and anxiety, doubt about the currency and authoritativeness of the documents being accessed was growing, and confusion related to clinical practice guidelines for the provision of safe, person centred care. At the same time, partner organizations and external provincial health teams without access to the organization's intranet required access to the same information, tools, and resources, which created additional pressure to find a solution. **Outcomes:** In response, an interprofessional team comprising of Policy, Library, Interprofessional Practice & Learning, and Communications, with approval through the COVID-19 Clinical Medical Advisory Committee (CMAC) and the Emergency Operations Centre (EOC), was struck to resolve this problem; Policy Office and Library Services leaders co-led the project. Due to the collaborative structure of this team, gaps and potential bottlenecks identified and addressed, and a content management solution was conceptualized, built, and implemented that remains at core of the province's COVID-19 response today. **Discussion:** Through this paper, we will share the finer points of this journey: mapping out efficient and sustainable practices, the bumps felt along the way, how early and constant user feedback was critical, our successes, where we are today, and where we are headed with this specific content management system.

### CP14. Single & ready to mingle: Forging connections & the silver lining of solo librarianship

Jeanna Hough
Halton Healthcare

**Introduction:** I've been a solo Librarian for 13 years. While difficult, I will look at how this allows a unique professional freedom and forging connections through program planning outside the traditional Library walls. One interwoven theme will be how we frame/view our circumstances and align our limitations/ resources, including how the Pandemic has offered additional opportunities. **Description:** I will summarize some partnerships/programs such as: ● External partnership with iMD: consumer health tool ● Public Library partnerships: Health literacy talks, Welcome Baby program for early health literacy ● Inter-hospital partnerships: With Holland-Bloorview in Toronto with their neurodevelopmental video game lending program for kids ● Department connections: managing a team of 25 PPE superusers during the Pandemic ● Internal hospital projects: Heading up Patient Education policy making ● Leading the education portfolio on the bedside entertainment terminals ● Integrating POC tools and Patient Education into our updated EMR. While these partnerships/programs are additional for a solo Librarian, they continue to reinforce the Library and Librarian as highly valued partners in the hospital. Additionally it increases the visibility as a leader and information expert, and improved usage statistics. **Outcomes:** I will touch on how the programs are going, look at numbers if applicable or available as well as discuss impacts to professional capacity. **Discussion:** The programs are ongoing and will be assessed further and used as jumping grounds for additional connection building and program planning. Discussion will also touch on the value add for a Librarian personally and professionally.

## CP15. Extending the Bramer Method with EndNote 20: Deduplicating with DOI

**Introduction:** Since comprehensive searching for knowledge syntheses requires searching in multiple databases with overlapping content, record deduplication is an important step in the review process. Manual deduplication is time-consuming, and although automated options exist, these are not always accurate or available. The method published by Bramer et al. in 2016 ('Bramer Method') combines manual and automated deduplication using the reference management software EndNote to systematically and reproducibly deduplicate records. **Description:** Researchers conducting knowledge syntheses at our institution were accustomed to having records deduplicated by library staff. However, due to staffing reductions, this service was unlikely to be sustainable as it was. Instead of cancelling the service, staff adapted the Bramer Method to deduplicate records by Digital Object Identifier (DOI) using EndNote 20. This presentation will outline the time-saving steps used to deduplicate database records in EndNote 20, as well as lessons learned during the process. **Outcomes:** While this extended method requires a few additional steps, the duration of the deduplication process has been reduced to almost half of the time taken using the original Bramer Method. **Discussion:** Record deduplication is a time-consuming process, and it may be challenging for library staff to offer this service amid staffing changes and increasing workloads. However, accurate and efficient deduplication is a useful skill that adds additional value to the information professional's role on a knowledge synthesis team.

## CP16. Pointers for preprints: Searching for medical preprints and comparing aggregators

Sarah C. McGill
CADTH

Introduction: Preprints share emerging research developments months before a final publication, and their production and dissemination exploded during the COVID-19 pandemic. Though preprints are not peer reviewed, they can provide important information about upcoming research and confirm research gaps. Since 2020, CADTH has added a preprint search for our COVID-19 reports and evidence syntheses. Purpose: We will discuss search approaches for preprints, including advising systematic review teams, managing citations, and selecting search aggregators. We will describe the approach for a recent long COVID scoping review that included preprints. Suggestions and Discussion: Where preprints are of importance, a preprint-specific aggregator should be added to the search rather than relying on traditional bibliographic databases because the latter currently have limited numbers of preprints. Preprint aggregators, such as Europe PMC, OSF Preprints, and preVIEW, compile multiple preprint servers and have individual advantages, such as free web-based platforms and advanced Boolean search, as well as disadvantages like variable options for limiting and saving searches. As preprints are plentiful but transitional, some developers are exploring user annotations or subject headings tagged by artificial intelligence. Even as COVID-19 evolves, preprint aggregators develop new search features. We will provide a comparison table to help decide which preprint aggregator is best for your search.

## CP17. Comparison of asynchronous online versus in-person library instruction methods for teaching literature searching

Sandra McKeown, Angélique Roy
Queen's University

**Objectives:** University instructors were abruptly thrust into an online learning environment at the beginning of the COVID-19 pandemic in 2020. The liaison librarian for graduate medicine at a Canadian university adapted an in-person library session on literature searching in bibliographic databases to a form of asynchronous online instruction during this period. Students returning to campus in 2022 presented an opportunity for the librarian to compare the effectiveness of the traditional in-person library session with this new form of online instruction. The primary objective of this study was to compare the effectiveness of traditional in-person library instruction with asynchronous online library instruction for teaching literature searching skills to students enrolled in a translational medicine graduate course. The secondary objective is to determine the students' perceived effectiveness of the different instruction methods and their attitudes towards different teaching formats for learning literature searching skills. **Methods:** Students enrolled in a translational medicine graduate course in Fall 2022 were invited to participate in this research study. 12 of 17 students agreed to participate and were randomly assigned to a control group (receiving in-person library instruction), or to an intervention group (receiving a link to a narrated PowerPoint video). Both instructional sessions were 80 minutes and included structured slide content and demonstrations in Ovid MEDLINE. Using Qualtrics online survey tool, participants completed one pre- and two post-tests containing the same 10 multiple choice questions to assess learning and retention, and a survey to evaluate their perception of the instruction received and their attitudes towards different instruction methods for literature searching. **Results:** The ability to analyze pre- and post-test scores was affected by missing data due to the low completion rates among the intervention group. We calculated the change between time points in pre- and post-test scores for each group and then compared the change between the two groups to account for knowledge scores at baseline. While the results were not statistically significant, the in-person group improved more than the video group from pre- to post-test 1, and from pre- to post-test 2. All 6 participants in the intervention group rated the pace of the narrated PowerPoint video as just right, compared to 4 out of 6 in the control group. 4 out of 6 participants in the intervention group found the instruction method mostly effective, and 2 of 6 somewhat effective, compared to 3 of 6 for both mostly effective and somewhat effective among the control group. Ratings for preferred instruction methods and the most important factors identified for learning about literature searching will be discussed. **Conclusions:** While many studies have investigated the effectiveness of in-person or online library instruction methods, few compare different instruction methods in a single study. This is the first study to randomly assign students to an intervention and control group for receiving library instruction on literature searching, and the first study to investigate the effectiveness of a narrated PowerPoint video as a form of asynchronous online instruction. However, the sample size for this study is very small and may not be generalizable to other graduation students receiving library instruction for literature searching.

## CP18. Evaluating the performance of RefWorks and Deduklick for de-duplicating references

Sandra McKeown, Zuhaib Mir
Queen's University

**Introduction:** There are various automated electronic methods for deduplicating references after searching multiple resources during the process of conducting an evidence synthesis including deduplication features in citation managers and systematic review tools. This research examines the performance of default algorithms for deduplicating references in RefWorks (using the revamped deduplication feature released by ProQuest in November 2022), Deduklick (introduced in 2022 as a stand-alone deduplication AI tool), and Systematic Review Accelerator (a suite of automation tools that includes a "Deduplicator" feature). **Methods:** Database search strategies from a previously conducted evidence synthesis were used to collect a heterogenous sample of references from the following Ovid databases: MEDLINE, Embase, PsycINFO and Cochrane CENTRAL. Manual abstraction of the sample of references was used to identify duplicates and develop a benchmark for comparing deduplicated references from RefWorks, Deduklick, and Systematic Review Accelerator. The number of false negative and false positive references generated by each program was determined to calculate accuracy, sensitivity, and specificity. **Results:** The performance of default settings for deduplicating references in RefWorks, Deduklick, and Systematic Review Accelerator will be presented and directly compared to other citation managers and systematic review software programs, which have been previously evaluated and reported on using the same sample of references and process of analysis. **Conclusions:** The performance of electronic methods for deduplicating references should be taken into consideration when conducting evidence syntheses to avoid unintentionally removing eligible studies, which could introduce bias. The cost and availability of programs that deduplicate references may limit and/or exclude their use among researchers, which may have implications for evidence synthesis quality.

### CP19. A tale of two studies: From research to action with researcher profile systems

Caroline Monnin, Justin Fuhr
University of Manitoba

**Introduction:** Researcher Profile Systems, such as ORCID, ResearchGate, Google Scholar Profiles, and ScopusID, are an integral part of the research life cycle to ensure accurate scientific output and discoverability. Researcher profiles support name disambiguation, accurate bibliometrics, and visibility. Health sciences and hospital librarians are important participants in advocating and supporting these tools to post-secondary and hospital communities. To improve our instruction and bibliometric services, it is important to understand which profiles are being used. **Methods:** Our study is comprised of two parts. First, we evaluated synchronous instructional sessions to determine which tools participants opted to use after attending. Second, we used a sample to evaluate use of researcher profiles at our institution, the University of Manitoba. After manually collecting publicly available data, we calculated the statistical correlation between profiles and discipline and rank. **Results:** Our health sciences faculty adoption rate is 72.2% for ORCID (+26.7% over reference value), 97.2% for ScopusID (+11.8%), 37.5% for Google Scholar Profiles (-1%), and 72.2% for ResearchGate (+12.1%). Health sciences faculty prefer ORCID and are 8.7x more likely to use it than humanities. ScopusID and ResearchGate are also popular. Use of ORCID and ScopusID increased after attending library workshops. **Discussion:** Health sciences staff, faculty, and researchers are busy and maintaining multiple profiles is challenging. Our results will inform library services at our institution and hopefully beyond. With the outcome of our case study, we can revise our instruction to explain the importance of complete and public profiles, develop tutorial and web content to explain discipline-specific choice of profile, with the goal of increasing rate of adoption.

### CP20. Credit where credit is due: A content analysis of librarians' roles in knowledge syntheses

Caroline Monnin, Mê-Linh Lê,
University of Manitoba

**Introduction:** Librarians play a vital role in knowledge synthesis (KS) research from constructing the search strategies to completing the data collection. Depending on the level of support librarians may be given co-authorship, an acknowledgement, or an in-text mention. While building upon previous work that benchmarks KS output at an institution, this work specifically examines KS at the University of Manitoba (UM) wherein our faculty are primary authors and if and how librarians were credited for their work.

**Methods:** We undertook a content analysis of all KS published between 2017-2022 by UM authors. We searched affiliation in PubMed and Scopus to retrieve 2,602 records. After screening, we had 696 records. From these records we determined the type of KS (e.g., rapid, scoping), the broad topic area (e.g., medicine, nursing, education) whether the primary authors were UM-affiliated, and the credited role of the librarian (if any). **Results:** We will calculate the type and number of KS completed during this period, the most popular disciplines, if the project used a librarian and if so, how the librarian was acknowledged. Results will be available in Spring 2023. **Discussion:** The information gathered in this research will be turned into action in three ways: 1) Advocating to our administration and faculty on the importance of this work with accurate data on the true scope of KS research at our institution; 2) Identifying [institution] affiliated authors who are doing KS research without the support of librarians and who may be amenable to outreach 3) Quantifying the level of credit librarians are getting for their KS work.

## CP21. It's broken, and we gotta fix it: Developing a new library service model in a multi-site hospital network

Zachary Osborne[1], Sabine Calleja[1], Michael Myers[2], Carolyn Ziegler[1]
[1]Unity Health Toronto; [2]Seneca College

**Introduction:** How does a library team of 6 support the research, training, and information needs of over 10,000 hospital staff across three different healthcare sites? The answer is: smarter, not harder. In 2022, planning started to determine a new model for library services to ensure outputs remain highly-valued and staff time is occupied meaningfully and equitably. This paper outlines the methods and project planning for a crucial initiative to reimagine the services and staffing at a multi-site hospital library system, proposing a new library service model (LSM). **Description:** An in-depth project charter was formulated to define the scope, deliverables, milestones, timelines, and stakeholders of the work. The project team organized deliverables into phases for information gathering, stakeholder engagement, data synthesis, and drafting recommendations. The work of the initial deliverables took shape as reviewing published and grey literature, an environmental scan of peer hospital institution libraries through structured interviews, gathering service statistics and benchmarks, and engaging with internal stakeholders to understand information needs and service gaps. It is anticipated the recommendations for a new proposed LSM will be available in April 2023, and if accepted will be implemented in 2024. **Outcomes:** High-level outcomes of this initiative will be shared at the time the paper is presented. Presenters will share reflections on the strengths and lessons learned of methods used to undertake this initiative. It is hoped other hospital libraries considering modifications to how they support their communities will benefit from the approaches and project planning employed in this experience. **Discussion:** This session will discuss the methods and approaches used to gather relevant information, and share preliminary details of the proposed new library service model.

## CP22. The ol' medical colouring book: Library outreach through play

Angélique Roy, Brendan Edwards,
Queen’s University

**Introduction:** As concerns for students' mental health rises, academic libraries remain essential in providing creative activities and services to help mitigate stress and anxiety (Bladek, 2021; Rose et al., 2015). While adult colouring books thrived in the mid-2010s across disciplines and markets (Blackburn & Chamley, 2016; Dresler & Perera, 2019; Garner et al. 2016), librarians at the Health Sciences and Rare Books libraries at a Canadian university recognized an opportunity to engage with students about library collections through a medical colouring book during the 2022 Fall semester. **Description:** The Ol' Medical Colouring Book was intended as a de-stressor activity during exams, featuring images from antiquarian anatomy and botany texts housed in special collections. It was designed primarily for Life Sciences and History of Medicine students by a second-year History student as part of an experiential learning internship, co-supervised by the librarians. Two three-hour pop-up events were held wherein the historical texts were displayed for students to browse and inquire about the history and provenance of the works. A unique hashtag was developed for promotion across online platforms. **Outcomes:** The pop-ups were received with overwhelming enthusiasm and the initial print run was distributed in under two hours of the first event which resulted in doubling the order for the second. An ISBN was obtained, and a copy was deposited in the university's institutional repository so views can be tracked. **Discussion:** This initiative, which began as a simple idea between two colleagues, speaks to the transformative potential of the arts and humanities in medical education. Learnings from successes and shortcomings will be analyzed to develop subsequent editions into a unique learning object through faculty consultation and collaboration.

## CP23. Embedding information literacy within a required undergraduate curriculum

Denise Smith, Stephanie Sanger
McMaster University

**Background:** The Health Sciences Library and the Bachelor of Health Sciences (BHSc) program at McMaster University worked together to build a multi-year information literacy (IL) curriculum embedded within the program under a suite of courses called Praxis Pathways. **Description:** The Praxis Pathways information literacy program is the focus of this presentation. The authors will describe the multi-year embedded IL curriculum, which is scaffolded to build both IL skills, such as database searching, and introduce students to key conceptual conversations in IL, production and dissemination. **Outcomes:** BHSc program graduates in 2023 will be the first to have completed all four years of the Praxis Pathways courses, including the IL program developed and delivered by the library. The authors will describe how the impact of the program will be evaluated qualitatively and quantitatively going forward. **Conclusion:** Embedded librarianship for multi-year, scaffolded IL education in undergraduate programs continues to be a rarity, despite the acknowledgement that one-shot instruction has several limitations. The authors present this program description to share how they embedded a for-credit IL curriculum in an undergraduate program that looks beyond the one-shot, skill-based tutorial and focuses on developing adaptive, information-literate lifelong learners.

## CP24. Measuring Institutional Research Impact with Wikipedia Citations

Denise Smith, Jack Young, Jennifer McKinnell
McMaster University

**Introduction:** Traditional research metrics tell us about a publication's or researcher's impact within their scholarly community, but they do not describe impact outside academia. Altmetrics fills this gap by tracking mentions in sources widely accessible to the public, including Wikipedia. Wikipedia is the most frequently accessed online health information resource and is well-positioned as a public health communication tool and knowledge translation tool. The authors aimed to explore their institution's health and medical research impact by analysing its presence in Wikipedia articles. **Methods:** In October 2022, a comprehensive database search was constructed in PubMed to retrieve clinical evidence syntheses published by at least one McMaster University author from 2017 to 2022, inclusive. Altmetics Explorer was queried using PubMed Identifiers and article titles to access metadata and Wikipedia citation data for 3,582 knowledge synthesis outputs. **Results:** 6% (n=219) of health evidence syntheses from the authors' institution were cited 568 times in 524 Wikipedia articles across 29 unique languages. 45% of citations appeared in English Wikipedia, suggesting a broad global reach of outputs. When adjusted for open access, 8% of McMaster University's health evidence syntheses appear in Wikipedia. A chi-square test confirmed a relationship between open-access publications and their presence in Wikipedia. **Conclusion:** Altmetrics Explorer is a valuable tool for exploring the impact of an institution's research outputs. Isolating altmetric data to focus on Wikipedia citations has value for institutions wishing to gain more insight into the global, community-level reach of its contributions to the latest health and medical evidence.

### CP25. Why is access to library resources so complicated? A case study of library technology

Kelly Thormodson, Ben Hoover
Penn State University

**Introduction:** The consolidation and growth of health systems in the US has had a major impact on associated academic libraries. The complications of this growth on libraries include collections, management and administration and most especially technology. This paper takes a look at specifically the technology issues faced by Penn State University's Harrell Library whose health system grew 400% in less than 7 years. Over the last 25 years, nearly all medical libraries content has switched to primarily electronic format. The standard being that access is provided via IP (Internet Protocol) while on campus and via a log in with an associated university/health center ID and password from off campus (through a VPN or proxy server). **Description/Methods:** Technology issues related to the growth of the health system were complicated by budget issues and thus caused a division in who was and was not allowed access. The library worked with IT to find solutions to how access was granted and authenticated to those who were covered by resource contracts. **Outcomes/Results:** Temporary solutions are in place for access to various groups but are not perfect. The solution to providing access to distinct groups involved making access to library information resources harder and more complicated for the end user. Frustration and complaints have increased from the communities the library serves. **Discussion:** While users are getting access to resources, there are complications still to be fixed. Other issues are at play that effect the technology, which is not always in the control of the library. The library continues to work to make the access to resources as uncomplicated as possible.

### CP26. Beyond the barriers: health information seeking behaviour of transgender identifying people in Nova Scotia, Canada

Francis Toole
Dalhousie University

**Introduction:** The purpose of this research project is to explore and understand the barriers that currently exist for transgender people in Nova Scotia, Canada when navigating adequate healthcare and how these individuals use other avenues of information gathering to meet their needs. **Methods:** Following ethics approval from Dalhousie University Research Ethics Board, this mixed methods study included interviews with transgender participants and a survey of current healthcare workers. The semi-structured interviews used the Information World Mapping (IWM) structure, having each participant engage in a creative drawing activity to share their stories and experiences within the healthcare system. Survey results from healthcare workers currently in the system provide further insight to transgender healthcare currently being provided. **Results:** Traditional healthcare settings, particularly emergency rooms, can be a place of misgendering for transgender people. The majority of interview participants relied more on social networks, social media and other internet based tools to gather information prior to appointments as opposed to coming to appointments to gather information. Overall, the survey responses confirmed a need for more systemic training, understanding and consideration of transgender issues, terminology and care.

Many survey respondents showed a care for these issues but felt a lack of support and identified underlying issues with the system itself. **Discussions and Recommendations:** This research indicates a gap in how transgender health information is available to the community and healthcare providers. This gap in service drives individuals to gather information online to find adequate information. Based on these findings improvements should be made, including: increased time for appointments, clearer guidelines and information regarding gender-affirming care, and further training for providers.

## CP27. Change-readiness scale for library managers: Development and analysis

**Introduction:** This study focuses on developing a scale that encompasses aspects of readiness to change by library management in dealing with the next normal scenario in libraries. It investigates measuring change readiness in terms of library service operations, workflows, administration, programs, and spaces, which can give an overview of a library institution's preparedness to meet new roles and expectations. **Methods:** The descriptive quantitative research was employed using a two-phase approach. It attempts to design an instrument referred to as Change-Readiness Instrument for Library Managers (CRILM), which was derived from an original self-developed questionnaire consisting of 20-item readiness-for-change attributes. The validity and reliability of CRILM were tested using Exploratory Factor Analysis (EFA) and Cronbach's Alpha. Sample size calculation was employed to determine the required number of subjects to participate in the study. Library managers who are head librarians from different types of libraries were the target samples. Content validation of the items was conducted for suitability and context. **Results:** CRILM was tested both valid and reliable. The initial results yielded from the respondent sample indicated that CRILM is deemed suitable for measuring change readiness among library managers and libraries. **Discussion:** The factor-analyzed items were grouped into three sub-scales based on their characteristics, namely: Flexibility in work and services, Learning support readiness and Adaptive to changing service environments. CRILM covered the major domains and constructs of change readiness in the context of a library setting. CRILM should be subjected to further testing and use by a wider target group to strengthen its usability and confidence level.

## CP28. Repositioning a college library in response to user needs: a case study.

**Introduction:** This case study will describe a transformational change in a health sciences library aimed to adjust library collections, services and physical and virtue spaces to the needs of diverse groups of users and the institutional strategic goals. It will provide practical strategies used in developing, implementing and promoting radical changes, reflect on challenges, and conclude with recommendations. **Methods:** The data for this case study was obtained through the use of a student satisfaction survey, consultations with internal stakeholders, and a literature search on current trends in academic health libraries. A thorough analysis of collected data helped identify important next steps in the evolution of the library and informed the library's strategic planning to make improvements. **Results:** Changes in key library domains (collections, services and space) were proposed and implemented in 2019-2022. This involved transition to mostly digital library, development and integration of an innovative instructional model into curriculum, re-purposing of a physical space, and a technological upgrade. The transformation led to increased traffic to digital resources, a major shift in the evidence-based practice instruction within the institution, and improved visibility and reputation of the library within and outside the institution. **Discussion:** Within a constantly changing academic environment, adaptability of library collections, services and spaces to changes in technology, user information needs and the institutional strategic priorities is viewed as a major contributing factor for both the library's and institutional success. The findings from this case study show that a clear vision of the library's purpose informed by user needs assessment, motivated library staff, effective promotion, and a creative collaboration with internal stakeholders help to unlock the library's value within an academic institution.

### CP29. How “open” are we? A content analysis of journal policies in library and information science

Marisa Tutt
Island Health

**Introduction:** Like the patrons we serve, library and information science (LIS) practitioners working outside of academia (e.g., hospital libraries) and/or in resource-limited settings, are facing barriers to participating in evidence-informed practice. Notably, subscription costs and article processing charges (APCs) are limiting and determining not only who can participate but how, what, and where knowledge is shared. Within the medical library context, most journals offer some sort of open access (OA). Outside of a handful of diamond OA journals (mostly association journals), however, many of these OA journals follow a hybrid or green model where large portions of content remain behind paywalls and cost prohibitive APCs and subscription fees restrict access and authorship. As part of the 2022-2023 Medical Library Association's Research Training Institute a research project has been developed to answer the question: how open are LIS journals? The goal of this study is to shine a light on LIS publishing practices to establish if libraries, given their support for OA, "practice what they preach." **Methods:** In this study, 'open' refers to whether or not the journals published OA content as opposed to offering the option to publish open. A web content analysis is underway as of writing this abstract. A list of peer-reviewed LIS journals has been compiled from a number of indexing sources in addition to practitioner developed lists. Characteristics of each journal are being recorded through an analysis of the journal websites including the percentage of OA articles published, type of publication models, and fees (subscription and APC). **Results:** Results are forthcoming. Preliminary results will be shared and discussed at CHLA 2023 with a focus on medical library journals.

### CP30. Subduing the 'moral panic': Sustaining a nuanced conversation about predatory publishing

Elizabeth Yates
Brock University

**Introduction:** Predatory publishing has long raised alarm bells among faculty, librarians and research administrators. Often falsely conflated with open access publishing as a whole, predatory publishing is painted as a grievous threat to the sanctity of scholarly research and a waste of research funding. However, the 'moral panic'* over predatory publishing may be unjustified. Equipping researchers to make informed decisions about publishing is a more sustainable approach. Building partnerships and sharing evidence about faculty publishing patterns can support this type of advocacy. **Description:** Librarians at institutions without subscriptions to costly citation analysis tools such as Scopus may find it difficult to analyze open access publishing patterns. However, freely available academic search tools such as The Lens can provide useful snapshots to guide education and support for researchers. Data from Brock University indicates that our faculty are overwhelmingly publishing in OA journals which would not typically be deemed predatory. Partnering with institutional research services via outreach and workshops has allowed the Library to share this data and other information about open access with key audiences of research administrators and faculty. **Outcomes:** This outreach helps the Library to guide researchers towards a more robust understanding of open access and scholarly publishing and away from reliance on problematic tools such as blacklists. In addition to helping authors make informed decisions about where to publish, such programming has boosted uptake for research consultations around publishing and open access. **Discussion:** The presenter will share tools and strategies for implementing this collaborative approach at other libraries. ***Houghton, F. (2022). Keep calm and carry on: moral panic, predatory publishers, peer review, and the emperor's new clothes. JMLA, 110(2), 233.

JOURNAL INFORMATION
==============================
NLM Title Abbreviation: J Can Health Libr Assoc
Journal ID: J Can Health Libr Assoc
NLM Journal ID: 101229936
Journal ID: JCHLA

The Journal of the Canadian Health Libraries Association

EISSN: 1708-6892
Publisher: Canadian Health Libraries Association / Association des bibliotèques de la santé du Canada

ARTICLE INFORMATION
==============================
Subjects: CP = Contributed Paper

CP1. Exploring the impact of automated indexing on completeness of MeSH terms

Amar-Zifkin Alexandre
Paquet Virginie
Ekmekjian Taline
Landry Tara
Université de Montréal

JOURNAL INFORMATION
==============================
NLM Title Abbreviation: J Can Health Libr Assoc
Journal ID: J Can Health Libr Assoc
NLM Journal ID: 101229936
Journal ID: JCHLA

The Journal of the Canadian Health Libraries Association

EISSN: 1708-6892
Publisher: Canadian Health Libraries Association / Association des bibliotèques de la santé du Canada

ARTICLE INFORMATION
==============================
Subjects: CP = Contributed Paper

CP2. Can GPT-3 tools accurately find and analyze articles for systematic reviews? A (very) preliminary assessment

Atwood Gary
University of Vermont

JOURNAL INFORMATION
==============================
NLM Title Abbreviation: J Can Health Libr Assoc
Journal ID: J Can Health Libr Assoc
NLM Journal ID: 101229936
Journal ID: JCHLA

The Journal of the Canadian Health Libraries Association

EISSN: 1708-6892
Publisher: Canadian Health Libraries Association / Association des bibliotèques de la santé du Canada

ARTICLE INFORMATION
==============================
Subjects: CP = Contributed Paper

CP3. Preliminary results of a longitudinal study of health information-seeking behaviour pre- and post-COVID

Bartlett Joan
Bowen-Ziecheck Aaron
McGill University

JOURNAL INFORMATION
==============================
NLM Title Abbreviation: J Can Health Libr Assoc
Journal ID: J Can Health Libr Assoc
NLM Journal ID: 101229936
Journal ID: JCHLA

The Journal of the Canadian Health Libraries Association

EISSN: 1708-6892
Publisher: Canadian Health Libraries Association / Association des bibliotèques de la santé du Canada

ARTICLE INFORMATION
==============================
Subjects: CP = Contributed Paper

CP4. Librarian teaching practices in support of knowledge syntheses projects: Preliminary results of an environmental scan

Bradley-Rideout Glyneva 1
Parker Robin 2
Quaiattini Andrea 3
Nevison Maggie 4
Sikora Lindsey 5
Fuller Kaitlin 6
1 Gerstein Science Information Centre, University of Toronto
2 Dalhousie University
3 McGill University
4 University of Toronto
5 University of Ottawa
6 St. Francis Xavier University

JOURNAL INFORMATION
==============================
NLM Title Abbreviation: J Can Health Libr Assoc
Journal ID: J Can Health Libr Assoc
NLM Journal ID: 101229936
Journal ID: JCHLA

The Journal of the Canadian Health Libraries Association

EISSN: 1708-6892
Publisher: Canadian Health Libraries Association / Association des bibliotèques de la santé du Canada

ARTICLE INFORMATION
==============================
Subjects: CP = Contributed Paper

CP5. Putting a sustainable systematic review into action

Chung Sunny
Scheinfeld Laurel
Koos Jessica
Saragossi Jamie
Stony Brook University

JOURNAL INFORMATION
==============================
NLM Title Abbreviation: J Can Health Libr Assoc
Journal ID: J Can Health Libr Assoc
NLM Journal ID: 101229936
Journal ID: JCHLA

The Journal of the Canadian Health Libraries Association

EISSN: 1708-6892
Publisher: Canadian Health Libraries Association / Association des bibliotèques de la santé du Canada

ARTICLE INFORMATION
==============================
Subjects: CP = Contributed Paper

CP6. An environmental scan of librarian involvement in systematic reviews and scoping reviews at the University of Ottawa

Farrell Peter
Cole Victoria
DaSilva Emily
Ly Valentina
Dehler Nicholas
Hofi Alain El
Sterling Evan
Rokolj Téa
Visintini Sarah
Labelle Patrick
Sikora Lindsay
University of Ottawa

JOURNAL INFORMATION
==============================
NLM Title Abbreviation: J Can Health Libr Assoc
Journal ID: J Can Health Libr Assoc
NLM Journal ID: 101229936
Journal ID: JCHLA

The Journal of the Canadian Health Libraries Association

EISSN: 1708-6892
Publisher: Canadian Health Libraries Association / Association des bibliotèques de la santé du Canada

ARTICLE INFORMATION
==============================
Subjects: CP = Contributed Paper

CP7. Advocating online for better knowledge translation in New Brunswick and beyond during the COVID-19 pandemic: I tried

Gadd Kathleen
Johnson Cheryl

JOURNAL INFORMATION
==============================
NLM Title Abbreviation: J Can Health Libr Assoc
Journal ID: J Can Health Libr Assoc
NLM Journal ID: 101229936
Journal ID: JCHLA

The Journal of the Canadian Health Libraries Association

EISSN: 1708-6892
Publisher: Canadian Health Libraries Association / Association des bibliotèques de la santé du Canada

ARTICLE INFORMATION
==============================
Subjects: CP = Contributed Paper

CP8. Perceptions and behaviours of first year health sciences undergraduate students conducting online research

Galbraith Susanna
McMaster University

JOURNAL INFORMATION
==============================
NLM Title Abbreviation: J Can Health Libr Assoc
Journal ID: J Can Health Libr Assoc
NLM Journal ID: 101229936
Journal ID: JCHLA

The Journal of the Canadian Health Libraries Association

EISSN: 1708-6892
Publisher: Canadian Health Libraries Association / Association des bibliotèques de la santé du Canada

ARTICLE INFORMATION
==============================
Subjects: CP = Contributed Paper

CP9. Advocating for space in a hospital: one library's experience

Gleeson Sarah
Lavoie Ashley
Fraser Health

JOURNAL INFORMATION
==============================
NLM Title Abbreviation: J Can Health Libr Assoc
Journal ID: J Can Health Libr Assoc
NLM Journal ID: 101229936
Journal ID: JCHLA

The Journal of the Canadian Health Libraries Association

EISSN: 1708-6892
Publisher: Canadian Health Libraries Association / Association des bibliotèques de la santé du Canada

ARTICLE INFORMATION
==============================
Subjects: CP = Contributed Paper

CP10. Factors and outcomes of collaboration in keeping up to date: An exploratory multiple case study

Granikov Vera 1
Bouthillier France 2
Pluye Pierre 3
1 Centre de recherche du CHUM
2 School of Information Studies, McGill University
3 Department of Family Medicine, McGill University

JOURNAL INFORMATION
==============================
NLM Title Abbreviation: J Can Health Libr Assoc
Journal ID: J Can Health Libr Assoc
NLM Journal ID: 101229936
Journal ID: JCHLA

The Journal of the Canadian Health Libraries Association

EISSN: 1708-6892
Publisher: Canadian Health Libraries Association / Association des bibliotèques de la santé du Canada

ARTICLE INFORMATION
==============================
Subjects: CP = Contributed Paper

CP11. Learning on the job: Using artificial intelligence and natural language processing to support rapid review methods

Hagerman Leah
Neil-Sztramko Sarah
Dobbins Maureen
The National Collaborating Centre for Methods and Tools

JOURNAL INFORMATION
==============================
NLM Title Abbreviation: J Can Health Libr Assoc
Journal ID: J Can Health Libr Assoc
NLM Journal ID: 101229936
Journal ID: JCHLA

The Journal of the Canadian Health Libraries Association

EISSN: 1708-6892
Publisher: Canadian Health Libraries Association / Association des bibliotèques de la santé du Canada

ARTICLE INFORMATION
==============================
Subjects: CP = Contributed Paper

CP12. Methods to support evidence-informed decision-making in public health: creation and evolution of a rapid review service

Hagerman Leah
Nayab Choudhry
Neil-Sztramko Sarah
Clark Emily
Snelling Susan
Traynor Robyn
Dobbins Maureen
The National Collaborating Centre for Methods and Tools

JOURNAL INFORMATION
==============================
NLM Title Abbreviation: J Can Health Libr Assoc
Journal ID: J Can Health Libr Assoc
NLM Journal ID: 101229936
Journal ID: JCHLA

The Journal of the Canadian Health Libraries Association

EISSN: 1708-6892
Publisher: Canadian Health Libraries Association / Association des bibliotèques de la santé du Canada

ARTICLE INFORMATION
==============================
Subjects: CP = Contributed Paper

CP13. Finding our way in a time of uncertainty: collaboration and the COVID-19 Hub

Hodder Joanne 1
Helliwell Michelle 1
Mercer Kate 1
Millen Chelsey 1
Whelan Noella 1
Daurie Ashley 1
MacDonald Leah 1
McLean Katie 1
Puxley Katie 2
1 Nova Scotia Health Authority
2 Nova Scotia College of Art & Design

JOURNAL INFORMATION
==============================
NLM Title Abbreviation: J Can Health Libr Assoc
Journal ID: J Can Health Libr Assoc
NLM Journal ID: 101229936
Journal ID: JCHLA

The Journal of the Canadian Health Libraries Association

EISSN: 1708-6892
Publisher: Canadian Health Libraries Association / Association des bibliotèques de la santé du Canada

ARTICLE INFORMATION
==============================
Subjects: CP = Contributed Paper

CP14. Single & ready to mingle: Forging connections & the silver lining of solo librarianship

Hough Jeanna
Halton Healthcare

JOURNAL INFORMATION
==============================
NLM Title Abbreviation: J Can Health Libr Assoc
Journal ID: J Can Health Libr Assoc
NLM Journal ID: 101229936
Journal ID: JCHLA

The Journal of the Canadian Health Libraries Association

EISSN: 1708-6892
Publisher: Canadian Health Libraries Association / Association des bibliotèques de la santé du Canada

ARTICLE INFORMATION
==============================
Subjects: CP = Contributed Paper

CP15. Extending the Bramer Method with EndNote 20: Deduplicating with DOI

Main Emilia
Sunnybrook Health Sciences Centre Jeanna Hough

JOURNAL INFORMATION
==============================
NLM Title Abbreviation: J Can Health Libr Assoc
Journal ID: J Can Health Libr Assoc
NLM Journal ID: 101229936
Journal ID: JCHLA

The Journal of the Canadian Health Libraries Association

EISSN: 1708-6892
Publisher: Canadian Health Libraries Association / Association des bibliotèques de la santé du Canada

ARTICLE INFORMATION
==============================
Subjects: CP = Contributed Paper

CP16. Pointers for preprints: Searching for medical preprints and comparing aggregators

McGill Sarah C.
CADTH

JOURNAL INFORMATION
==============================
NLM Title Abbreviation: J Can Health Libr Assoc
Journal ID: J Can Health Libr Assoc
NLM Journal ID: 101229936
Journal ID: JCHLA

The Journal of the Canadian Health Libraries Association

EISSN: 1708-6892
Publisher: Canadian Health Libraries Association / Association des bibliotèques de la santé du Canada

ARTICLE INFORMATION
==============================
Subjects: CP = Contributed Paper

CP17. Comparison of asynchronous online versus in-person library instruction methods for teaching literature searching

McKeown Sandra
Roy Angélique
Queen’s University

JOURNAL INFORMATION
==============================
NLM Title Abbreviation: J Can Health Libr Assoc
Journal ID: J Can Health Libr Assoc
NLM Journal ID: 101229936
Journal ID: JCHLA

The Journal of the Canadian Health Libraries Association

EISSN: 1708-6892
Publisher: Canadian Health Libraries Association / Association des bibliotèques de la santé du Canada

ARTICLE INFORMATION
==============================
Subjects: CP = Contributed Paper

CP18. Evaluating the performance of RefWorks and Deduklick for de-duplicating references

McKeown Sandra
Mir Zuhaib
Queen’s University

JOURNAL INFORMATION
==============================
NLM Title Abbreviation: J Can Health Libr Assoc
Journal ID: J Can Health Libr Assoc
NLM Journal ID: 101229936
Journal ID: JCHLA

The Journal of the Canadian Health Libraries Association

EISSN: 1708-6892
Publisher: Canadian Health Libraries Association / Association des bibliotèques de la santé du Canada

ARTICLE INFORMATION
==============================
Subjects: CP = Contributed Paper

CP19. A tale of two studies: From research to action with researcher profile systems

Monnin Caroline
Fuhr Justin
University of Manitoba

JOURNAL INFORMATION
==============================
NLM Title Abbreviation: J Can Health Libr Assoc
Journal ID: J Can Health Libr Assoc
NLM Journal ID: 101229936
Journal ID: JCHLA

The Journal of the Canadian Health Libraries Association

EISSN: 1708-6892
Publisher: Canadian Health Libraries Association / Association des bibliotèques de la santé du Canada

ARTICLE INFORMATION
==============================
Subjects: CP = Contributed Paper

CP20. Credit where credit is due: A content analysis of librarians' roles in knowledge syntheses

Monnin Caroline
Lê Mê-Linh
University of Manitoba

JOURNAL INFORMATION
==============================
NLM Title Abbreviation: J Can Health Libr Assoc
Journal ID: J Can Health Libr Assoc
NLM Journal ID: 101229936
Journal ID: JCHLA

The Journal of the Canadian Health Libraries Association

EISSN: 1708-6892
Publisher: Canadian Health Libraries Association / Association des bibliotèques de la santé du Canada

ARTICLE INFORMATION
==============================
Subjects: CP = Contributed Paper

CP21. It's broken, and we gotta fix it: Developing a new library service model in a multi-site hospital network

Osborne Zachary 1
Calleja Sabine 1
Myers Michael 2
Ziegler Carolyn 1
1 Unity Health Toronto
2 Seneca College

JOURNAL INFORMATION
==============================
NLM Title Abbreviation: J Can Health Libr Assoc
Journal ID: J Can Health Libr Assoc
NLM Journal ID: 101229936
Journal ID: JCHLA

The Journal of the Canadian Health Libraries Association

EISSN: 1708-6892
Publisher: Canadian Health Libraries Association / Association des bibliotèques de la santé du Canada

ARTICLE INFORMATION
==============================
Subjects: CP = Contributed Paper

CP22. The ol' medical colouring book: Library outreach through play

Roy Angélique
Edwards Brendan
Queen’s University

JOURNAL INFORMATION
==============================
NLM Title Abbreviation: J Can Health Libr Assoc
Journal ID: J Can Health Libr Assoc
NLM Journal ID: 101229936
Journal ID: JCHLA

The Journal of the Canadian Health Libraries Association

EISSN: 1708-6892
Publisher: Canadian Health Libraries Association / Association des bibliotèques de la santé du Canada

ARTICLE INFORMATION
==============================
Subjects: CP = Contributed Paper

CP23. Embedding information literacy within a required undergraduate curriculum

Smith Denise
Sanger Stephanie
McMaster University

JOURNAL INFORMATION
==============================
NLM Title Abbreviation: J Can Health Libr Assoc
Journal ID: J Can Health Libr Assoc
NLM Journal ID: 101229936
Journal ID: JCHLA

The Journal of the Canadian Health Libraries Association

EISSN: 1708-6892
Publisher: Canadian Health Libraries Association / Association des bibliotèques de la santé du Canada

ARTICLE INFORMATION
==============================
Subjects: CP = Contributed Paper

CP24. Measuring Institutional Research Impact with Wikipedia Citations

Smith Denise
Young Jack
McKinnell Jennifer
McMaster University

JOURNAL INFORMATION
==============================
NLM Title Abbreviation: J Can Health Libr Assoc
Journal ID: J Can Health Libr Assoc
NLM Journal ID: 101229936
Journal ID: JCHLA

The Journal of the Canadian Health Libraries Association

EISSN: 1708-6892
Publisher: Canadian Health Libraries Association / Association des bibliotèques de la santé du Canada

ARTICLE INFORMATION
==============================
Subjects: CP = Contributed Paper

CP25. Why is access to library resources so complicated? A case study of library technology

Thormodson Kelly
Hoover Ben
Penn State University

JOURNAL INFORMATION
==============================
NLM Title Abbreviation: J Can Health Libr Assoc
Journal ID: J Can Health Libr Assoc
NLM Journal ID: 101229936
Journal ID: JCHLA

The Journal of the Canadian Health Libraries Association

EISSN: 1708-6892
Publisher: Canadian Health Libraries Association / Association des bibliotèques de la santé du Canada

ARTICLE INFORMATION
==============================
Subjects: CP = Contributed Paper

CP26. Beyond the barriers: health information seeking behaviour of transgender identifying people in Nova Scotia, Canada

Toole Francis
Dalhousie University

JOURNAL INFORMATION
==============================
NLM Title Abbreviation: J Can Health Libr Assoc
Journal ID: J Can Health Libr Assoc
NLM Journal ID: 101229936
Journal ID: JCHLA

The Journal of the Canadian Health Libraries Association

EISSN: 1708-6892
Publisher: Canadian Health Libraries Association / Association des bibliotèques de la santé du Canada

ARTICLE INFORMATION
==============================
Subjects: CP = Contributed Paper

CP27. Change-readiness scale for library managers: Development and analysis

Torres Efren Jr 1
Abrigo Christine 2
1 De La Salle Medical and Health Sciences Institute
2 De La Salle University

JOURNAL INFORMATION
==============================
NLM Title Abbreviation: J Can Health Libr Assoc
Journal ID: J Can Health Libr Assoc
NLM Journal ID: 101229936
Journal ID: JCHLA

The Journal of the Canadian Health Libraries Association

EISSN: 1708-6892
Publisher: Canadian Health Libraries Association / Association des bibliotèques de la santé du Canada

ARTICLE INFORMATION
==============================
Subjects: CP = Contributed Paper

CP28. Repositioning a college library in response to user needs: a case study

Tukhareli Natalia
Canadian Chiropractic Memorial College in Toronto

JOURNAL INFORMATION
==============================
NLM Title Abbreviation: J Can Health Libr Assoc
Journal ID: J Can Health Libr Assoc
NLM Journal ID: 101229936
Journal ID: JCHLA

The Journal of the Canadian Health Libraries Association

EISSN: 1708-6892
Publisher: Canadian Health Libraries Association / Association des bibliotèques de la santé du Canada

ARTICLE INFORMATION
==============================
Subjects: CP = Contributed Paper

CP29. How “open” are we? A content analysis of journal policies in library and information science

Tutt Marisa
Island Health

JOURNAL INFORMATION
==============================
NLM Title Abbreviation: J Can Health Libr Assoc
Journal ID: J Can Health Libr Assoc
NLM Journal ID: 101229936
Journal ID: JCHLA

The Journal of the Canadian Health Libraries Association

EISSN: 1708-6892
Publisher: Canadian Health Libraries Association / Association des bibliotèques de la santé du Canada

ARTICLE INFORMATION
==============================
Subjects: CP = Contributed Paper

CP30. Subduing the 'moral panic': Sustaining a nuanced conversation about predatory publishing

Yates Elizabeth
Brock University

